# CD8^+^ T-cell encephalitis mimicking PRES in AIDS: a case report

**DOI:** 10.1186/s12883-020-01756-7

**Published:** 2020-05-12

**Authors:** Mayu Ishiguro, Yuji Ueno, Yuta Ishiguro, Masashi Takanashi, Kenji Murai, Guillaume Taieb, Kensuke Daida, Akimitsu Suda, Kazumasa Yokoyama, Toshio Naito, Nobutaka Hattori

**Affiliations:** 1 Department of Neurology, Juntendo University Faculty of Medicine, 2-1-1 Hongo, Bunkyo-ku, Tokyo, 113-8421 Japan; 2 Department of General Medicine, Juntendo University Faculty of Medicine, Tokyo, Japan; 3 Department of Neurology, University of Montpellie, Faculty of Medicine, Montpellier, France

**Keywords:** CD8^+^ encephalitis, AIDS, Immune reconstitution inflammatory syndrome, Posterior reversible encephalopathy syndrome, Case report

## Abstract

**Background:**

Diverse mechanisms including infections, autoimmune inflammatory reactions, neoplasms, and degeneration are involved in the central nervous system in cases of acquired immune deficiency syndrome. In such cases, it is difficult to determine the precise pathogenesis by radiological examination and laboratory testing.

**Case presentation:**

We report a 37-year-old Japanese woman who had untreated hypertension and gender identity disorder and had been taking testosterone injections since she was 19 years old. She developed a headache and visual field deficits together with elevated blood pressure. According to radiological findings, she was initially suspected as having posterior reversible encephalopathy syndrome in the right parieto-occipital lobe with reversible cerebral vasoconstriction syndrome. Human immunodeficiency virus antibody was positive and the CD4^+^ T-lymphocyte count was 140 cells/μl. Therefore, antiretroviral therapy was started. Antiretroviral therapy suppressed the activity of acquired immune deficiency syndrome but worsened her visual symptoms and expanding radiological lesions. Brain biopsy led to the diagnosis of CD8^+^ encephalitis, and she also fulfilled the diagnosis of paradoxical immune reconstitution inflammatory syndrome. Corticosteroid therapy alleviated her symptoms.

**Conclusions:**

This is a rare case of CD8^+^ encephalitis, with an exacerbation owing to paradoxical immune reconstitution inflammatory syndrome after antiretroviral therapy, which radiologically mimicked posterior reversible encephalopathy syndrome. Corticosteroid therapy was effective; thus, it is important to provide a pathological diagnosis in such cases.

## Background

A variety of comorbidities including infections, autoimmune inflammatory reactions, neoplasms, and degeneration are involved in the central nervous system (CNS) in acquired immune deficiency syndrome (AIDS) patients [1–3]. It is difficult to determine the pathogenesis of such CNS complications by radiological examination and laboratory testing; therefore, brain biopsy is necessary [4]. Here, we report the case of a patient who developed bilateral posterior lesions together with elevated blood pressure, mimicking posterior reversible encephalopathy syndrome (PRES) [5]. However, the case showed recurrent and progressive symptoms, and brain biopsy was carried out.

## Case presentation

**Fig. 1:**
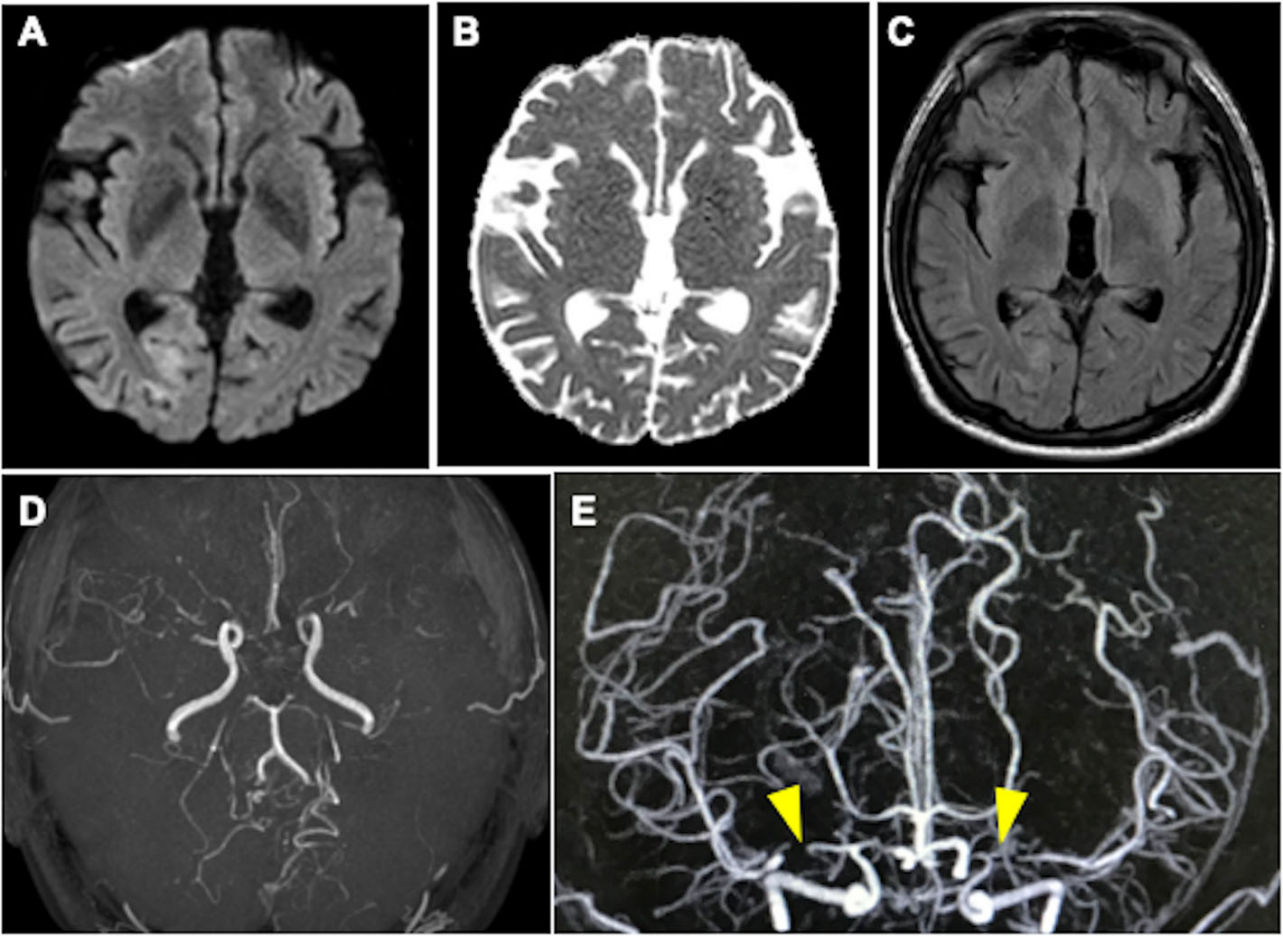
Radiological observations on the first admission **a–c**. Brain magnetic resonance imaging (MRI) on the first admission shows a hyperintense lesion in the right parieto-occipital lobe on diffusion-weighted imaging (**a**), apparent diffusion coefficient map (**b**), and fluid-attenuated inversion recovery (**c**). **d**. MR angiography shows stenosis in bilateral middle cerebral arteries (MCAs). **e.** Three-dimensional contrast-enhanced angiography revealed occlusions in bilateral MCAs (yellow arrowheads)

**Fig. 2:**
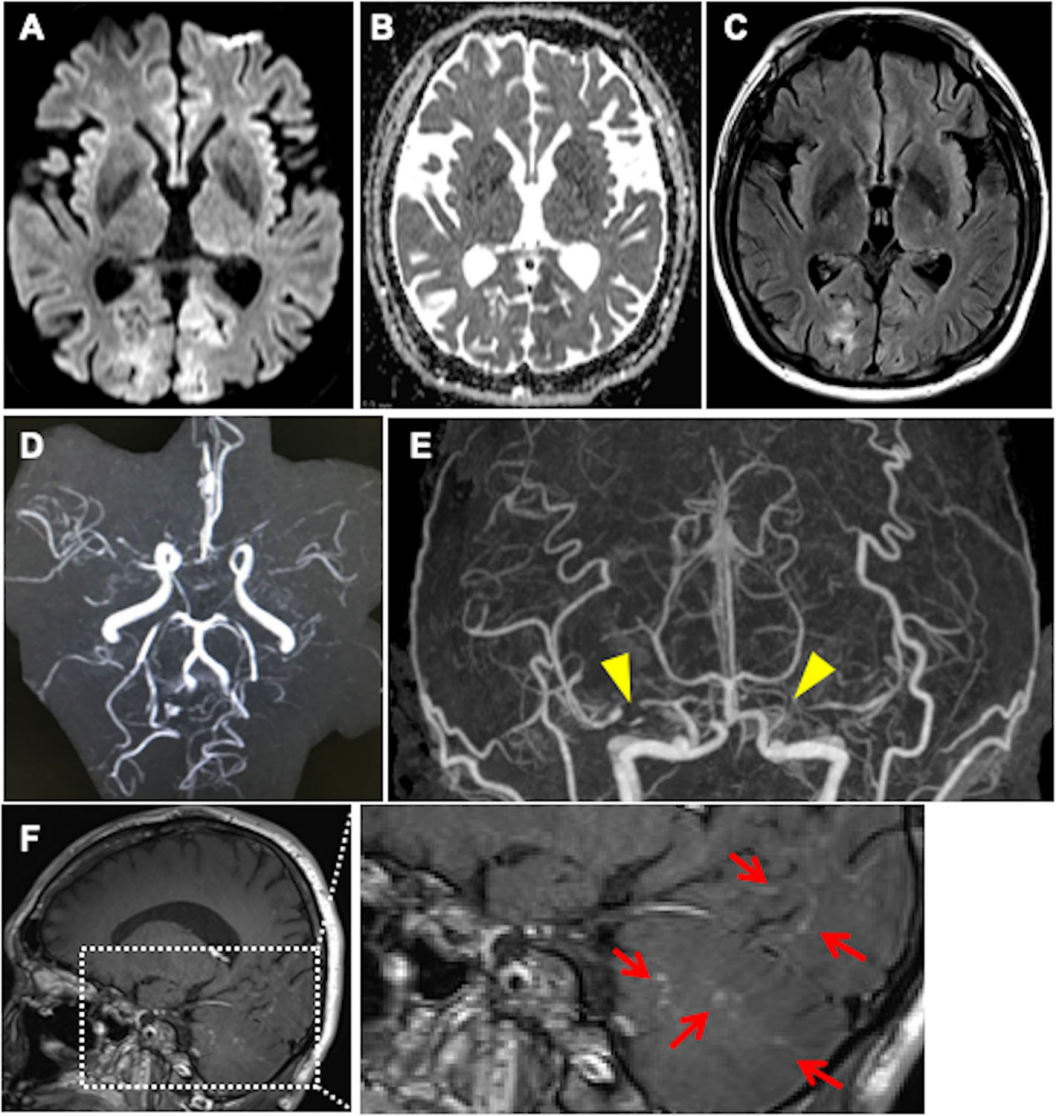
Radiological observations on the second admission a**–c**. Brain magnetic resonance imaging (MRI) on the second admission shows hyperintense lesions in the bilateral occipital lobe on diffusion-weighted imaging (**a**), apparent diffusion coefficient map (**b**), and fluid-attenuated inversion recovery (**c**). **d**. MR angiography shows stenosis in bilateral middle cerebral arteries (MCAs). **e.** Three-dimensional contrast-enhanced angiography revealed occlusions in bilateral MCAs (yellow arrowheads). **f**. Contrast-enhanced MRI shows multiple punctate and linear gadolinium-enhanced lesions (red arrows) in the occipital and temporal lobes and cerebellum

**Fig. 3:**
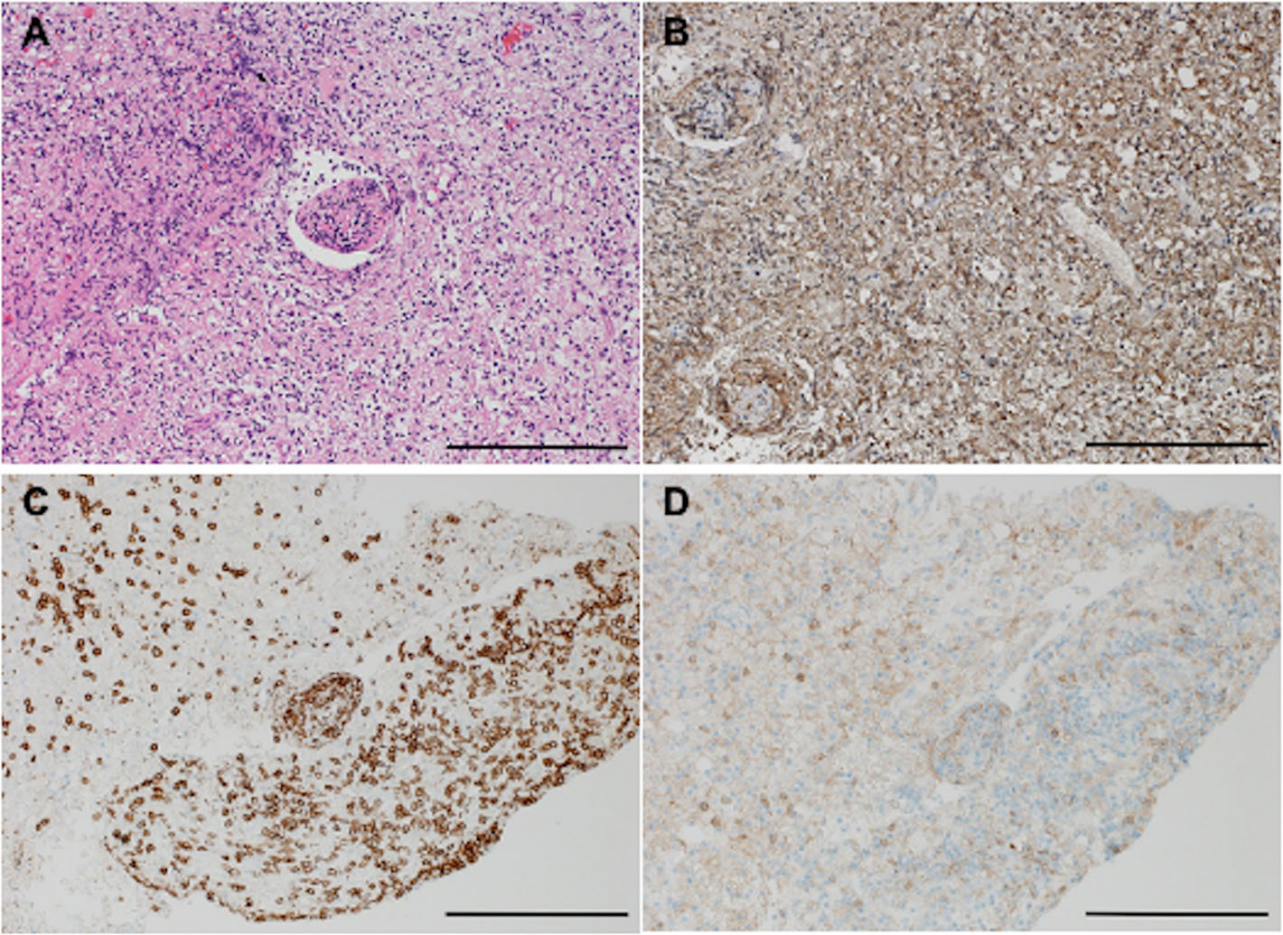
Pathological study from brain biopsy samples a–d. Pathological findings from brain biopsy samples in the right occipital lobe show severe lymphocytic infiltration with vasculitis (**a**, hematoxylin and eosin) and activated microglia (**b**, Iba-1) in the white matter. Most lymphocytes were CD8 T-cells (**c**, CD8), and CD4 T-cells were scarce (**d**, CD4). Scale bars = 200 μm

A 37-year-old Japanese woman had a history of gender identity disorder from childhood and had taken testosterone injections once every 2 weeks since she was 19 years old. She also had untreated high blood pressure. She had worked in the sex industry and had a tattoo on her right arm. In May 2017, she developed a headache and visual field deficits together with elevated blood pressure and was referred to our hospital. On admission, her blood pressure was 165/105 mmHg with regular heart rhythm. She was alert and well oriented. She had left homonymous hemianopia. Brain magnetic resonance imaging (MRI) showed a hyperintense lesion in the right parieto-occipital lobe on diffusion-weighted imaging (DWI), apparent diffusion coefficient (ADC) map, and fluid-attenuated inversion recovery (FLAIR) (Fig. 1a–c), which were not enhanced by contrast with gadolinium. MR angiography (MRA) showed steno-occlusive lesions in bilateral middle cerebral arteries (MCAs) (Fig. 1d). Three-dimensional contrast-enhanced angiography revealed occlusions of bilateral MCAs (Fig. 1e). She was initially suspected as having PRES related to reversible cerebral vasoconstriction syndrome (RCVS) and received treatment with an antihypertensive drug and 100 mg of aspirin. Routine blood testing showed the patient was HIV-1 antibody-positive. The CD4^+^ T-cell count was 140 cells/μl and the HIV viral load detected by PCR was 330,000 copies/ml. She underwent lumbar puncture, and no pleocytosis was found. Furthermore, PCR for herpes simplex virus (HSV), varicella-zoster virus (VZV), and JC virus in cerebrospinal fluid was negative. She had also developed pneumocystis pneumonia when she was diagnosed with AIDS. Antiretroviral therapy (ART) comprising dolutegravir sodium, emtricitabine, and tenofovir alafenamide fumarate was initiated, and she was discharged from the hospital. Two weeks later, she suffered a severe headache and worsening of visual disturbance in bilateral eyes. Her blood pressure was 153/93 mmHg and her visual acuities were finger counting. MRI showed the hyperintense lesion had expanded to bilateral posterior hemispheres (Fig. 2a–c). Stenotic lesions in bilateral MCAs remained on MRA and three-dimensional contrast-enhanced angiography (Fig. 2d, e). The CD4^+^ T-cell count at readmission was 189 cells/μl and HIV viral load was 94 copies/ml, indicating that AIDS activity was alleviated after ART. Although she was initially treated with edaravone, a free radical scavenger, and antihypertensive agents after readmission, her visual acuities fluctuated and contrast-enhanced MRI showed multiple punctate and linear gadolinium-enhanced lesions in the occipital and temporal lobes and the cerebellum (Fig. 2f). Brain biopsy was performed from the right occipital lobe. Histopathology showed severe tissue destruction, astrocytic gliosis, microglial activation, and vasculitis with marked lymphocytic infiltration in the cerebral white matter in the absence of multinucleated giant cells and lymphoma cells (Fig. 3a, b). Infiltrated lymphocytes were mostly CD8^+^ T-lymphocytes, while CD4^+^ T-lymphocytes were scarce (Fig. 3c, d). We finally diagnosed her as having CD8^+^ encephalitis, with an exacerbation caused by immune reconstitution inflammatory syndrome (IRIS) after ART. After brain biopsy, the patient was treated with 1000 mg of methylprednisolone intravenously for 3 consecutive days followed by 0.5 mg/kg/day of prednisolone. Her visual acuities and headache improved after corticosteroid treatment.

## Discussion and conclusions

The present patient, who had gender identity disorder and was taking testosterone injections, developed a headache, high blood pressure, progressive focal neurological deficits and showed posterior hyperintense lesions on DWI/ADC/FLAIR and multifocal intracranial artery stenosis on MRA, which was likely radiological findings of PRES together with RCVS at the first admission. It has been reported that PRES can occur in AIDS patients [6]. After ART, the patient’s symptoms worsened and radiological findings showed focal hyperintensity on T2/FLAIR imaging with punctate and curvilinear gadolinium enhancements, which are characteristic of CD8^+^ encephalitis, progressive multifocal leukoencephalopathy (PML), and lymphoma recognized in HIV patients [1–4, 7]. Interestingly, brain biopsy in our case revealed CD8^+^ encephalitis; thus, brain biopsy should be considered in AIDS patients when the clinical course is progressive and radiological studies are insufficient.

CD8^+^ encephalitis has been characterized as a progressive encephalopathy that occurs in HIV-infected patients with clinical features of headache, cognitive impairment, seizure, and reactivity to corticosteroid therapy [4, 8]. In particular, a previous histopathological study clearly demonstrated infiltration of CD8^+^ T-cells and microglia in perivascular areas and parenchyma, indicating CD8^+^ encephalitis. In addition, findings excluded diagnoses of PML owing to lacking JC virus inclusions, HIV encephalitis related to multinuclear giant cells, and lymphoma due to the absence of abnormal proliferated cells, as well as infiltration of CD3^+^ and CD4^+^ T-cells in chronic lymphocytic inflammation with pontine perivascular enhancement responsive to steroids [2, 3, 9]. Furthermore, ART increased the CD4^+^ T-cell count and significantly reduced the HIV viral load, and occipital lesions on MRI expanded, indicating development of immune reconstitution in the brain; therefore, IRIS also might be involved in the pathogenesis of our case [1]. Our case developed a visual field defect as the initial symptom and right occipital lesion before ART, and fulfilled the diagnostic criteria for paradoxical IRIS [1]. Generally, AIDS patients with IRIS have concomitant infectious encephalitis or encephalopathy such as PML caused by JC virus, cryptococcal meningitis, VZV encephalitis, or tuberculosis. However, a previous case series demonstrated that few HIV cases, which were PCR-negative for HSV, VZV, and JC virus, developed CD8^+^ encephalitis and IRIS caused by noninfectious pathogens, which was consistent with our case [7, 8, 10]. These common mechanisms may be attributed to unbalanced reconstitution that proliferative CD8^+^ cytotoxic lymphocytes in the absence of CD4^+^ lymphocytes in CNS can be deleterious, despite the increase in systemic CD4^+^ lymphocytes after ART [8].

Importantly, our case exhibited bilateral MCA stenotic lesions on MRA, which led us to initially suspect PRES with RCVS. Whether CD8^+^ encephalitis with IRIS involves large intracranial arteries is presently unknown. RCVS is characterized by headache, stenotic changes in multiple intracranial large arteries for 1–3 months, and could simultaneously occur with PRES. [11] Alternatively, angiitis related to HIV, atherosclerotic changes owing to long-term hypertension, and administration of testosterone might be related to stenosis [12]. However, radiological observations after 3 months and pathological alterations in MCA were not investigated in our case; therefore, precise mechanisms remain unknown.

To our knowledge, this is a rare case of CD8^+^ encephalitis with an exacerbation owing to paradoxical IRIS after ART, which radiologically mimicked PRES. Corticosteroid therapy was effective; thus, it is important to provide a pathological diagnosis in such cases. However, involvement of cerebral large arteries is pathologically unknown, and further investigation is warranted.

## Data Availability

All the data supporting our findings are provided within the manuscript.

## Abbreviations

ADC: Apparent diffusion coefficient
AIDS: Acquired immune deficiency syndrome
ART: Antiretroviral therapy
CNS: Central nervous system
DWI: Diffusion-weighted imaging
FLAIR: Fluid-attenuated inversion recovery
IRIS: Immune reconstitution inflammatory syndrome
MCAs: Middle cerebral arteries
MRA: Magnetic resonance angiography
MRI: Magnetic resonance imaging
PCA: Posterior cerebral artery
PML: Progressive multifocal leukoencephalopathy
PRES: Posterior reversible encephalopathy syndrome
RCVS: Reversible cerebral vasoconstriction syndrome
